# Urinary Trivalent Methylated Arsenic Species in a Population Chronically Exposed to Inorganic Arsenic

**DOI:** 10.1289/ehp.7519

**Published:** 2004-11-22

**Authors:** Olga L. Valenzuela, Victor H. Borja-Aburto, Gonzalo G. Garcia-Vargas, Martha B. Cruz-Gonzalez, Eliud A. Garcia-Montalvo, Emma S. Calderon-Aranda, Luz M. Del Razo

**Affiliations:** ^1^Seccion de Toxicología, Cinvestav-IPN, México DF, México; ^2^Salud en el Trabajo, Instituto Mexicano del Seguro Social, México DF, México; ^3^Facultad de Medicina, Universidad Juárez del Estado de Durango, Gómez Palacio, Durango, México; ^4^Servicios de Salud del Estado de Hidalgo, Pachuca, México

**Keywords:** arsenic, arsenic skin lesions, arsenic speciation, hyperkeratosis, hyperpigmentation, hypopigmentation, metabolism, methylation, trivalent arsenic, trivalent methylarsenic species, urine metabolites

## Abstract

Chronic exposure to inorganic arsenic (iAs) has been associated with increased risk of various forms of cancer and of noncancerous diseases. Metabolic conversions of iAs that yield highly toxic and genotoxic methylarsonite (MAs^III^) and dimethylarsinite (DMAs^III^) may play a significant role in determining the extent and character of toxic and cancer-promoting effects of iAs exposure. In this study we examined the relationship between urinary profiles of MAs^III^ and DMAs^III^ and skin lesion markers of iAs toxicity in individuals exposed to iAs in drinking water. The study subjects were recruited among the residents of an endemic region of central Mexico. Drinking-water reservoirs in this region are heavily contaminated with iAs. Previous studies carried out in the local populations have found an increased incidence of pathologies, primarily skin lesions, that are characteristic of arseniasis. The goal of this study was to investigate the urinary profiles for the trivalent and pentavalent As metabolites in both high- and low-iAs–exposed subjects. Notably, methylated trivalent arsenicals were detected in 98% of analyzed urine samples. On average, the major metabolite, DMAs^III^, represented 49% of total urinary As, followed by DMAs^V^ (23.7%), iAs^V^ (8.6%), iAs^III^ (8.5%), MAs^III^ (7.4%), and MAs^V^ (2.8%). More important, the average MAs^III^ concentration was significantly higher in the urine of exposed individuals with skin lesions compared with those who drank iAs-contaminated water but had no skin lesions. These data suggest that urinary levels of MAs^III^, the most toxic species among identified metabolites of iAs, may serve as an indicator to identify individuals with increased susceptibility to toxic and cancer-promoting effects of arseniasis.

Arsenic is a ubiquitous element found in several forms in foods and environmental media such as soil, air, and water; the predominant form in drinking water is inorganic As (iAs), which is both highly toxic and readily bioavailable. iAs is a recognized carcinogen in humans [National Research Council (NRC) 2001]. Chronic ingestion of iAs-contaminated drinking water is therefore considered the major pathway behind the risk to human health. It has been estimated that 200 million people worldwide are at risk from health effects associated with high concentrations of As in their drinking water (NRC 2001). Several other regions in the world are exposed to levels above the maximum permissible limit recommended by the World Health Organization (WHO 1993).

In humans, the chronic ingestion of iAs (> 500 μg/day As) has been associated with cardiovascular, nervous, hepatic, and renal alterations and diabetes mellitus as well as cancer of the skin, bladder, lung, liver, and prostate [Agency for Toxic Substances and Disease Registry (ATSDR) 2000]. Characteristic features of arseniasis include skin manifestations, such as hyperpigmentation, hypopigmentation, and hyperkeratosis on the palms and soles, and skin cancer at later stages (Cebrian et al. 1983; Schwartz 1997; Tseng et al. 1968).

In humans, the mechanism by which iAs exerts its toxic effects is very complex because its metabolism involves at least five metabolites that can exert toxic effects. The scheme for the stepwise conversion of arsenite (iAs^III^) into mono-, di-, and trimethylated products is as follows:


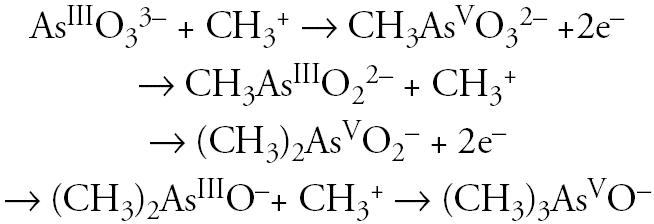


Briefly, the metabolic process is carried out in two processes: *a*) the reactions of reduction that convert the pentavalent species to trivalency, and *b*) reactions of oxidative methylations where iAs is converted to mono-, di-, and trimethyl arsenic forms (MAs, DMAs, and TMAsO, respectively). Thus, both pentavalent methylarsenic (MAs^V^) and trivalent methylarsenic (MAs^III^) forms are intermediates or products of this pathway (Lin et al. 2002; Thomas et al. 2004).

Using *S*-adenosylmethionine as a methyl group donor, As methyltransferase (Cyt19, EC 2.1.1.138) has been shown to catalyze reactions, reduction and oxidative methylation, in rodents and humans (Waters et al. 2004). Other studies have shown the capacity of two mammalian proteins to reduce iAs^V^, glutathione *S*-transferase omega (GST Ω, EC 2.5.1.18) (Zakharyan et al. 2001) and the purine nucleoside phosphorylase (PNP, EC 2.4.2.1) (Nemeti et al. 2003; Radabaugh et al. 2002).

Urinary As is generally regarded as the most reliable indicator of recent exposure to iAs and is used as the main biomarker of exposure (Mushak and Crocetti 1995). In addition, the urinary profiles of iAs metabolites have frequently been used in epidemiologic studies to assess the capacity of exposed individuals to methylate iAs. During almost 20 years, the methylation of iAs has been generally evaluated using urinary measurement of iAs (III + V), MAs (III + V), and DMAs (III + V) in people exposed to As. Nevertheless, the differentiation of the trivalent intermediaries of As metabolism is important because the trivalent methylated arsenicals, MAs^III^ and DMAs^III^, are more potent than either iAs^III^ or iAs^V^ in cytotoxicity (Styblo et al. 2000), genotoxicity (Mass et al. 2001; Nesnow et al. 2002), and inhibition of enzymes with antioxidative functions (Lin et al. 2001; Styblo et al. 1997). Therefore, the formation of MAs^III^ and DMAs^III^ in the methylation pathway for iAs may play a significant role in the induction of toxic effects associated with exposures to iAs.

The goal of this study was to assess the urinary pattern of As methylation, including trivalent methylated metabolites, in an As-endemic population using freshly collected samples analyzed as soon as possible to avoid the oxidation of MAs^III^ and DMAs^III^, even at temperatures < 0°C (Del Razo et al. 2001; Gong et al. 2001). Additionally, we compared the pattern of urinary trivalent methylated metabolites between persons with and without skin lesions associated with arsenicism in an endemic Mexican area.

## Materials and Methods

### Site selection.

Study subjects were residents of Zimapan in the state of Hidalgo, an area located in the central part of Mexico, approximately 220 km from Mexico City. It has been a mining district since the 16th century. By 1810 there were 40 smelters operating in and around Zimapan (Garcia and Querol 1991). There have been no active smelters in Zimapan since the 1940s; however, tailing piles from the flotation process have accumulated in Zimapan for > 60 years. Tailings are sediments resulting from settling of ore’s wastes. Some rocks from the Zimapan Valley with higher than world average iAs concentrations for the rock type (2,550–21,400 mg/kg) were found in all the tailings (Mendez and Armienta 2003). Most of the current exposure occurs outside the Zimapan basin, but old tailings near the edge of the town are still an iAs pollution source. Two major pathways contaminate ground-water with iAs: *a*) iAs dissolved from ore and other minerals in the mining district can be transported through fractures in the limestone (mainly arsenopyrite) to water sources, and *b*) rainwater leaches through surrounding mine tailing piles (Armienta et al. 1997a). The National Water Commission of Mexico found high iAs concentrations in many of the wells in 1992. Water samples collected from springs and drilled wells (~ 180 m total depth) presented levels between 21 and 1,100 μg As/L (Comisión Nacional del Agua 1992).

In 1999, the municipality closed one of the wells connected to the municipal water system that contained the highest iAs concentration (1,100 μg/L). This action reduced the average iAs concentration in the municipal water from 580 to 350 μg/L. However, > 40% of the valley residents in this area are not connected to this municipal supply and rely on local springs and *norias* (bucket-wheel wells) for their potable water, and some of these sources are still heavily polluted with iAs.

### Subject selection.

We conducted a cross-sectional study with 104 participants who lived in areas where their drinking water normally contained iAs, 76 Zimapan residents exposed to ≥ 50 μg/L iAs and 28 individuals exposed to ≤ 10 μg/L iAs (controls), in accordance with the regulations of the Ethical Committee of the Faculty of Medicine, Juarez University of Durango. Subjects were recruited through door-to-door contact. They had to be at least 15 years of age and live in the town for the previous 2 years. Before enrollment in the study, each participant read and signed an informed consent form. Subjects were interviewed by trained interviewers regarding general characteristics with emphasis in personal habits, history and habits of water consumption, smoking habits, and medical, occupational, and residential histories. They underwent physical examination looking for typical dermatologic signs of arseniasis. These signs of arseniasis were evaluated by medical health care physicians, who were blind to time and level of exposure for each subject at the time of physical examination. The physicians have been evaluating dermatologic signs of arseniasis in Mexico for about 10 years. Participants were asked to exclude seafood from their diets for the preceding 4 days. Individuals who had received drugs with well-defined organ toxicity within the past 4 months or were suffering chronic alcoholism were excluded. Each family’s drinking water was analyzed for total As (TAs) concentration. The final decision on study eligibility was based on the measurement of TAs concentration in the household water source, and approximately 50% of the iAs-high exposed group presented with at least one skin sign of arseniasis, such as hypo/hyperpigmentation, palmoplantar hyperkeratosis, and ulcerative lesions as described by Yeh (1973).

### Exposure assessment.

We estimated the total liters of drinking water consumed per day by each subject on the basis of subjects’ statements. Daily estimates of As consumption were calculated as the product of the number of liters consumed per day and the As concentration in the subject’s drinking water source. Cumulative exposure to iAs or time-weighted iAs exposure (TWE) was calculated using the duration of exposure, the number of liters consumed per day, and the historical As concentration reported by the Mexican National Water Commission from 1992 to the present time (Garcia-Vargas et al. 1994).

### Chemicals.

Arsenic acid disodium salt (Na_2_HAs^V^O_4_) and sodium *m*-arsenite (NaAs^III^O_2_), both > 99% pure, were obtained from Sigma Chemical Co. (St. Louis, MO, USA). Methylarsonic acid (MAs^V^) disodium salt [CH_3_As^V^O(ONa)_2_; 99% pure] was obtained from Ventron (Danvers, MA, USA), and dimethylarsinic acid [DMAs^V^; as (CH_3_)_2_As^V^O(OH); 98% pure] was obtained from Strem (Newburyport, MA, USA). The trivalent methylated arsenicals methyloxoarsine (MAs^III^O; CH_3_As^III^O) and iododimethylarsine of DMAs^III^ [DMAs^III^I; (CH_3_)_2_As^III^I] were synthesized by W.R. Cullen (University of British Columbia, Vancouver, British Columbia, Canada) using previously described methods (Cullen et al. 1984; Styblo et al. 1997). Identity and purity of the synthesized arsenicals were confirmed using ^1^H nuclear magnetic resonance, mass spectrometry, and hydride generation–atomic absorption spectrophotometry (HG-AAS) as previously described (Hughes et al. 2000). In aqueous solutions, MAs^III^O and DMAs^III^I are presumed to form MAs^III^ and DMAs^III^, respectively. Working standards of these arsenicals that contained 1 μg/mL As were prepared daily from stock solutions. Sodium borohydride (NaBH_4_) was obtained from EM Science (Gibbstown, NJ, USA). Tris hydrochloride was purchased from J.T. Baker (Phillipsburg, NJ, USA). Creatinine kits were purchased from Randox (San Diego CA, USA). All other chemicals used were at least analytical grade. Standard reference material (SRM) water (SRM 1463c) and urine [SRM 2670; National Institute of Standards and Technology (NIST), Gaithersburg, MD, USA] were used for quality control of TAs in water and urine analysis, respectively.

### Drinking-water collection and processing.

Tap water samples were collected in the homes of potential subject families using acid-washed containers transported to the site of the study by the investigators. We collected a total of 91 water samples from 80 households (more than one sample was obtained from each household if participants used different sources of water to drink and cook). Water samples were stored at −20°C until subsequent assay. Water samples were transported to the Cinvestav-IPN laboratories in Mexico City for TAs analysis. TAs was determined by HG-AAS using a PerkinElmer 3100 spectrometer (PerkinElmer, Norwalk, CT, USA), equipped with a FIAS-200 flow injection atomic spectroscopy system as reported previously (Del Razo et al. 1990). All measurements were made using an As electrodeless discharge lamp. SRM 1463c was used for quality control of TAs in water analysis. The certified TAs concentration in SRM 1463c is 82.1 ± 1.2 μg/L. Replicate analyses of this SRM using the method described above gave concentrations of 82.7 ± 1.7 μg/L, which is in good agreement with the certified value.

### Urine collection and processing.

After clinical exploration, all participants were scheduled for urine sample collection each third day over 3 weeks in groups of 10–12 individuals each time. Subjects were seen between 0700 and 0800 hr at the local health center, where urine spot samples were collected with a minimum of contamination in 250-mL polypropylene containers that we provided. Urine samples were immediately frozen in dry ice. To prevent oxidation of unstable trivalent methylated arsenicals, frozen urine samples were immediately transported to Cinvestav-IPN laboratory and analyzed within 6 hr after collection.

A pH-specific HG-AAS has been optimized to permit simultaneous analysis of all known metabolites of iAs, including iAs^III^, iAs^V^, MAs^III^, MAs^V^, DMAs^III^, and DMAs^V^ in urine (Del Razo et al. 2001). This method is based on a pH-specific generation of hydrides from tri- and pentavalent iAs, MAs, and DMAs with subsequent chromatography and determination of As contents in HG-AAS. The HG-AAS apparatus was based on the design of Crecelius et al. (1986). For hydride generation at ≤ pH 2, 1 mL sample urine, 5 mL deionized water, and 1 mL 6 M hydrochloric acid (HCl) were placed into the reaction vessel. This mixture had a final pH of 1–2. For hydride generation at pH 6, 1 mL sample urine, 5 mL deionized water, 1 mL 2.5 M Tris-HCl, and 0.06 M NaOH buffer, pH 6, were placed into the reaction vessel. This mixture had a final pH of approximately 6. At either pH, thorough mixing of the contents of the reaction vessel was followed by injection into the reaction vessel of 1 mL of a 4% solution of NaBH_4_ in 0.02 M NaOH. Cold-trapped arsines were released from the U-tube by its removal from the liquid nitrogen and application of heat, for separation of the later arsines for a gradient of temperature.

We used SRM 2670 to validate analysis of TAs; SRM 2670 consists of two bottles of urine—one containing an elevated concentration of As and one containing a normal concentration. The certified TAs concentration in the elevated urine is 480 ± 100 μg/L. As in normal urine is not certified; however, the NIST provides a reference value of 60 μg/L. Replicate analyses of these SRMs using the method described above give concentrations of 507 ± 17 μg/L and 64 ± 5 μg/L, respectively, which are in good agreement with the certified and reference values. The TAs concentration in urine samples reported in this article is the sum of the concentrations of iAs^III^, iAs^V^, MAs^III^, MAs^V^, DMAs^III^, and DMAs^V^.

### Creatinine in urine.

Urinary creatinine was measured by the Jaffe reaction using a Randox commercial kit. Arsenical species concentrations in urine were corrected for creatinine concentration as an indication of urine dilution.

### Statistical methods.

Data analysis was carried out using Stata 8.0 statistical software (Stata Corp., College Station, TX, USA). Arsenical values were transformed to a log scale in order to calculate means and range, to perform statistical comparisons between groups, and to evaluate potential confounding factors. We used Mann-Whitney tests to compare urinary As metabolites among exposed groups with and without lesions. Potential confounding risk factors evaluated included age, sex, sunlight exposure, and TWE.

## Results

Eighty families with a total of 104 participants completed the sampling protocol (Table 1). Because of the lack of good job opportunities in this area, most of the young men emigrate out of the country; in consequence, most of the subjects (90%) were female. The concentration of TAs in home drinking water of study participants ranged from 1 to 1,054 μg/L. In 24 homes (30%), the subjects drank bottled water in addition to municipal water.

Urine samples from both controls and individuals chronically exposed to high iAs by consumption of drinking water containing this metalloid were analyzed to determine the concentrations of iAs^III^, MAs^III^, and DMAs^III^. Arsenical values were adjusted by creatinine concentration. Urinary creatinine measurements ranged from 105 to 3,230 mg/L, with an average of 595 mg/L. Average urinary concentrations of trivalent and pentavalent As species in the total study group are shown in Table 2. Methylated trivalent arsenicals were detected in 98% of analyzed urine samples. In addition, trivalent arsenicals (iAs^III^ + MAs^III^ + DMAs^III^) were the predominant species in urine samples (65%). On average, the major metabolite, DMAs^III^, represented 49% of total urinary As, followed by DMAs^V^ (23.7%), iAs^V^ (8.6%), iAs^III^ (8.5%), MAs^III^ (7.4%), and MAs^V^ (2.8%).

Cutaneous signs of arsenicism were observed in 55 individuals from the group exposed to ≥ 50 μg/L As in drinking water. The type and proportion of cutaneous signs observed in participants of this study are shown in Table 3. Hyperkeratosis in the palm or sole was the most frequent skin sign of arsenicism (56.6%).

Table 4 summarizes the average arsenical concentrations according to the level of As exposure and the presence of skin lesions. Interestingly, in the high-As-exposure group, subjects presenting cutaneous signs had significant increases in the concentration of MAs^III^. In addition, the average of relative proportion of urinary MAs^III^ was marginally higher in exposed individuals with skin lesions compared with those who drank iAs-contaminated water but had no skin lesions (Table 5).

The conditional logistic regression model was based on 76 subjects exposed to ≥ 50 μg/L As in drinking water; this model was adjusted by age, sex, and TWE. The risk of occurrence of arseniasis related to both absolute and relative quantity of MAs^III^ was significant (*p* < 0.008 and < 0.004, respectively). The odds ratios (OR) for the subjects having hyperkeratosis plantar was estimated to be 1.06 [95% confidence interval (CI), 1.03–1.19] for concentration of MAs^III^ and 1.22 (95% CI, 1.07–1.44) for relative proportion of MAs^III^. Even though DMAs^III^ was the main species found in urine, neither its concentration nor its relative proportion was associated with the risk of As skin lesions (data not shown).

Another variable, independent of iAs metabolites, that was significantly associated with the presence of hyperkeratosis plantar (*p* = 0.003) in the group who drank water containing As > 50 μg/L was the lifetime iAs exposure, estimated as TWE (OR = 1.20; 95% CI, 1.06–1.35).

## Discussion

### MAs^III^ and DMAs^III^ in urine.

The analysis of urinary trivalent methylated metabolites of iAs using freshly collected urine samples, within 6 hr of collection to reduce differences in handling among samples and to minimize the extent of oxidation of trivalent arsenicals before analysis, allowed detection of the presence of MAs^III^ and DMAs^III^ in 98% of the urine collected, even in urine samples from subjects with low As exposure (≤ 10 μg/L in drinking water; Table 4). The optimization of As speciation techniques has only recently permitted analysis of oxidation states of As in methylated metabolites. Initial studies using the optimized techniques detected small amounts of MAs^III^ and/or DMAs^III^ in urine from residents exposed to iAs in drinking water in several geographical regions, including Romania (Aposhian et al. 2000), Inner Mongolia (Le et al. 2000), Mexico (Del Razo et al. 2001), and West Bengal (Mandal et al. 2001). However, most of these studies analyzed urine samples that were stored for an extended time after collection. Because MAs^III^ and DMAs^III^ are rapidly oxidized even at temperatures < 0°C (Del Razo et al. 2001; Gong et al. 2001), these studies probably underestimated the concentrations of these metabolites. In contrast, analyses of freshly collected urine samples in this study showed that methylated trivalent arsenicals (MAs^III^ and DMAs^III^) are in fact prevalent urinary metabolites of iAs (Table 2). DMAs^III^ also was detected only when fresh void urine was collected from DMAs^V^-fed rats (Cohen et al. 2002). Csanaky and Gregus (2002) reported that intravenously injected iAs was excreted mainly as MAs^III^, and Suzuki et al. (2001) reported that this excreted MAs^III^ was conjugated by glutathione [MAs^III^(GS)_2_] in rat bile.

Urinary detection of methylated and dimethylated arsenicals containing As in both oxidation states indicates that metabolism of As involves changes in the oxidation state of As during methylation. It is likely that interactions of trivalent iAs metabolites with proteins and other cellular constituents are responsible for retention and toxic effects of As in tissues of animals and humans exposed to iAs. A study with a population chronically exposed to iAs from drinking water indicated that methylated arsenicals are also retained in tissues (Aposhian et al 1997, 2000). These As-exposed individuals were treated with an As chelator, 2,3-dimercaptopropane-1-sulfonic acid (DMPS), which resulted in a massive release of MAs, including MAs^III^, in urine, suggesting that DMPS mobilized tissue depots of iAs.

### Skin lesions and trivalent methylated metabolites of iAs.

Skin keratosis and changes in skin pigmentation are two hallmark signs of arseniasis. Many residents of the Zimapan area had skin lesions related to iAs exposure (Armienta et al. 1997b) (Table 3). According to the historical values of As in drinking water, most of the families who participated in this study were exposed to extremely high As concentrations at least from 1992 to 1999. After this time, the level of concentration of As in water has been decreased because the municipality closed one of the wells connected to the municipal water system that contained the highest iAs concentration (1,100 μg/L). In addition, several residents previously drank bottled water with normal values of As instead of drinking water from the municipal system. This fact can explain the great prevalence of dermatoxicity, despite the moderate concentration of arsenicals in urine observed in the present study (Table 4).

We also assessed the profile of iAs metabolites on dermatoxicity (Table 3). Previous studies in other regions, such as the Lagunera Region in Mexico (Del Razo et al. 1997) and Taiwan (Yu et al. 2000), showed that subjects with arseniasis were more likely to have a higher concentration of MAs in urine than were exposed individuals who had did not have arseniasis; at that time, the speciated arsenical concentrations in urine were made without mentioning their oxidation state. Recent advent of techniques for speciation of As has helped establish the speciation of As according to oxidation states.

Importantly, trivalent methylated metabolites, especially DMAs^III^, were not stable in human urine and oxidized quickly to yield pentavalent MAs^V^ and DMAs^V^ (Del Razo et al. 2001). As noted above, these samples were analyzed within 6 hr of collection to reduce differences among samples in handling and to minimize the extent of oxidation of As^III^ before analysis.

Interestingly, in our study the iAs-exposed individuals bearing skin signs of arsenicism had significantly higher urinary relative proportions and concentrations of MAs^III^. These data suggest that a high output of MAs^III^ in urine may predict increased susceptibility to arseniasis.

MAs^III^ and DMAs^III^ have been reported to be highly toxic in mammalian cells (Styblo et al. 2000, 2002) and genotoxic (Mass et al. 2001; Nesnow et al. 2002). The reason for the high toxicity of methylated trivalent arsenicals has not been adequately explained, except that methylated trivalent arsenicals exert genotoxicity via reactive oxygen species (Nesnow et al. 2002). Although the comparative toxicity of MAs^III^(GS)_2_ was about nine times higher than that of iAs^III^, the accumulation rate of MAs^III^(GS)_2_ was 40 times higher than that of iAs^III^. These results suggest that MAs^III^(GS)_2_ was more toxic than iAs^III^, at least in part due to the more efficient accumulation of As in cells (Hirano et al. 2004). Moreover, Vega et al. (2001) showed that normal human epidermal keratinocytes exposed to low doses (0.001–0.01 μM) of MAs^III^ stimulated expression of certain proinflammatory cytokines and growth factors that are critical to maintaining homeostasis and barrier integrity in the skin, suggesting that the overexpression of these products can lead the skin pathologic processes.

Another possible mechanism for higher toxicity of trivalent arsenicals compared with the corresponding pentavalent forms is that trivalent species have a higher affinity for thiol compounds (Shiobara et al. 2001).

In light of these observations, biomethylation of iAs, a process yielding toxic trivalent methylated metabolites, appears to be a mechanism of activation of As as a toxin and possibly as a carcinogen. Because of the adverse biologic effects of these metabolites, the analysis of urinary MAs^III^ may serve as an effective tool for the evaluation of health risks associated with exposure to iAs.

There were no significant associations between other confounding risk factors, such as duration of sunlight, and skin lesions (data not shown).

Another important factor associated with the presence of As skin lesions was the lifetime exposure of As evaluated as TWE. Table 1 shows the significant difference between this variable between exposed As subjects, demonstrating that the magnitude of exposure is directly related to the presence of skin lesions. In other words, the basic principle of dose–response relationship was fulfilled for the presence of As skin lesions.

The major limitations of this study are the small sample population and the fact that most of the participants were female. Previous reports have indicated that age, dose, pregnancy, and sex are among factors that affect the urinary profiles of iAs metabolites (Del Razo et al. 1997; Hughes et al. 2000; Yu et al. 2000). Results obtained in this study may not generalize to males or to the entire population.

This study provides novel data on the pattern of trivalent and pentavalent metabolites of iAs clearance in fresh urine from a population exposed to iAs in drinking water. Unlike other studies, in which urine samples were stored for several weeks before analysis, this study shows that MAs^III^ and DMAs^III^ in urine are predominant As species compared with their corresponding pentavalent arsenicals.

MAs^III^, the most potent toxicant in the entire metabolic pathway of iAs, could be mainly responsible for the toxic and carcinogenic effects of iAs exposure, and its detection and quantification in human populations can assist in risk assessment and could be the cause of As carcinogenesis. Our findings support the view that the extent and character of adverse effects associated with iAs exposures are at least in part determined by the rate of the formation and by the composition of iAs metabolites.

Further research in other populations with arseniasis is needed to confirm the potential relationship between the concentrations of MAs^III^ in biologic samples from human populations and the little-understood etiology of hyperkeratosis, skin hypo- or hyperpigmentation, and cancer that can result from chronic iAs exposure.

## Figures and Tables

**Table 1:** Demographics of the study population.

	Low iAs exposure	High iAs exposure			
Characteristics	Control	Without skin lesions	With skin lesions
No. of subjects	28	21	55
Sex*^a^*			
Male	2 (7.1)	1 (4.8)	7 (14.6)
Female	26 (92.9)	20 (95.2)	48 (85.4)
Age (years)	35 (18–50)	35 (21–49)	35 (15–51)
Sunlight exposure (hours)	2.3 (0–8)	2.2 (0–8)	2.8 (0–9)
TAs concentration in drinking water (μg/L)	1.6 (1–6)	117 (50–1,504)	115 (50–658)
Duration of iAs exposure (years)*^b^*	26 (4–50)	21 (4–49)	26 (4–51)
TWE (mg)	0.01 (0.01–0.06)	5.8 (0.02–16.3)	9.3 (0.23–26.9)*

Values are mean (range) except where noted.

a Values are mean (%).

b Duration of well water consumption.

* Statistically significant difference (*p* < 0.05) between the exposed individuals with and without skin lesions.

**Table 2:** Concentration and relative proportion of As species in residents of the Zimapan area (*n* = 104).

iAs metabolite in urine	Concentration (μg/g creatinine)	Relative proportion (%)
iAs^V^	5.84 (1–65.5)	8.6
iAs^III^	4.46 (0.1–172.3)	8.5
MAs^V^	1.45 (0.1–28.3)	2.8
MAs^III^	4.93 (0.1–101.9)	7.4
DMAs^V^	14.56 (1–710)	23.7
DMAs^III^	30.75 (0.1–506.3)	49
TAs	84.85 (9.1–1398.1)	100

Concentration of metabolites of As in urine are reported as geometric mean (range).

**Table 3:** Distribution of skin lesion in iAs-exposed subjects (*n* = 76).

	Frequency [no. (%)]		
Skin lesion	With lesion	w/o lesion
Hypopigmentation	36 (47.4)	40 (52.6)
Hyperpigmentation	29 (38.2)	47 (61.8)
Hypo-/hyperpigmentation	9 (11.8)	67 (88.2)
Hyperkeratosis on the palms	33 (43.4)	43 (56.6)
Hyperkeratosis on the soles	30 (39.5)	46 (60.5)
Hyperkeratosis on the palms or soles	43 (56.6)	33 (43.4)
Keratosis on the trunk	17 (22.4)	59 (77.6)
Cutaneous horns	4 (5.3)	72 (94.7)
Bowen’s disease	1 (1.3)	75 (98.7)
Squamous cell carcinoma	1 (1.3)	75 (98.7)

w/o, without. Frequency was calculated for 76 subjects exposed to ≥ 50 μg/L As in drinking water.

**Table 4:** Urinary pattern of iAs species in humans exposed to As through drinking water in the Zimapan area, according to level exposition and the presence of As skin lesions (*n* = 104).

	Metabolite concentration of iAs in urine (μg/g creatinine)			
As species	Control (*n* = 28)	Exposed, without lesions (*n* = 21)	Exposed, with lesions (*n* = 55)
IAs^V^	3.6 (1.0–10.5)	6.1 (2.0–37.2)	8.2 (2.4–65.5)
IAs^III^	1.6 (0.1–9.7)	6.9 (1.5–101.6)	6.3 (0.3–172.3)
MAs^V^	0.6 (0.1–5.5)	1.8 (0.3–16.6)	2.0 (0.1–28.3)
MAs^III^	2.2 (0.4–9.6)	4.8 (0.1–24.9)	7.5 (0.2–101.9)*
DMAs^V^	7.4 (1.8–38.5)	15.9 (1.0–226.5)	19.8 (1.0–710.1)
DMAs^III^	7.9 (0.1–65.4)	48.1 (2.2–206)	51.9 (1.4–506.3)
TAs	33.3 (9.1–106)	116 (61.2–371.7)	121.2 (51.9–1,398)

Values shown are geometric mean (range).

* Statistically significant difference (*p* < 0.05) between the exposed individuals with and without skin lesions (by Mann-Whitney test).

**Table 5:** Comparison of mean percentage of As species in urine among As-exposed subjects with and without skin lesions.

	Percent As-exposed subjects		
As species	Without lesions (*n* = 21)	With lesions (*n* = 55)
iAs^V^	6.5	7.9
iAs^III^	10.9	8.0
MAs^V^	3.5	2.3
MAs^III^	5.9	7.7*
DMAs^V^	21.5	23.1
DMAs^III^	51.7	51.0

* Statistically marginal difference (*p* = 0.072) between the exposed individuals with and without skin lesions (by Mann-Whitney test).
